# Cellulose-Acetate-Based Films Modified with Ag_2_O and ZnS as Nanocomposites for Highly Controlling Biological Behavior for Wound Healing Applications

**DOI:** 10.3390/ma16020777

**Published:** 2023-01-12

**Authors:** Amjad F. Alharthi, Mohamed Gouda, Mai M. Khalaf, Abraham Elmushyakhi, Manal F. Abou Taleb, Hany M. Abd El-Lateef

**Affiliations:** 1 Department of Chemistry, College of Science, King Faisal University, Al-Ahsa 31982, Saudi Arabia; 2 Chemistry Department, Faculty of Science, Sohag University, Sohag 82524, Egypt; 3 Department of Mechanical Engineering, College of Engineering, Northern Border University, Arar 91431, Saudi Arabia; 4 Department of Chemistry, College of Science and Humanities, Prince Sattam Bin Abdulaziz University, Al-Kharj 11942, Saudi Arabia; 5 Department of Polymer Chemistry, National Center for Radiation Research and Technology (NCRRT), Egyptian Atomic Energy Authority, Nasr City, Cairo 11762, Egypt

**Keywords:** wound healing, metal oxide nanocomposites, cellulose acetate, biomedical materials

## Abstract

For wound healing, functional films with certain physicochemical and biological properties are needed. Thus, the current work aimed to fabricate multifunctional materials comprising metal oxide nanoparticles loaded with an efficient polymer to be used as dressing material. A composite containing polymeric phases of cellulose acetate (CA) blended with zinc sulfide (ZnS), silver oxide (Ag_2_O), and graphene oxide (GO) was successfully synthesized. The prepared composite crystallinity was studied using the X-ray diffraction technique (XRD). Further, the functional groups and the elemental analysis were investigated using Fourier-transform infrared spectroscopy (FTIR) and energy-dispersive X-ray spectroscopy (EDX). Furthermore, the surface morphology was studied using scanning electron microscopy (SEM) to obtain the shape and size of particles. SEM showed that the particles were formed in wide distribution in the range of 18–915 nm with an average size of 235 nm for Ag_2_O/ZnS/GO/CA. The particle size of Ag_2_O in the CA film was in the range between 19 and 648 nm with an average size of 216 nm, while the particle size of ZnS in CA was in the range of 12–991 nm with an average age particle size of 158 mm. In addition, EDX, based on SEM investigation, detected high carbon and oxygen quantities at around 94.21% of the composite. The contact angle decreased and reached 26.28° ± 2.12° in Ag_2_O/ZnS/CA. Furthermore, thermogravimetric analysis (TGA) was used to investigate the thermal stability, and the composition was thermally stable until 300 °C. Moreover, the cell viability of “normal lung cells” reached 102.66% in vitro at a concentration of 1250 µg/mL. The antibacterial activity of Ag_2_O/ZnS/GO/CA was also detected against *E. coli* with a zone of inhibition reaching 17.7 ± 0.5 mm. Therefore, the composite can be used in biomedical applications due to its biocompatibility and antibacterial activity.

## 1. Introduction

Tissue engineering is an essential modern field specializing in treating or replacing degenerated or damaged tissues, such as bone tissues, instead of traditional clinical methods [1,2]. Conventional techniques have many drawbacks, such as immune system rejection, donor site scarcity, and microbial infection risks [2]. Further, polymers have vast choices to be used in tissue engineering applications [3]. The polymers can be from a natural or synthetic origin, and many have densities close to the biological tissues [4]. Therefore, they are used as films and under biomaterial classification. However, synthetic polymers such as polycaprolactone and polylactic acid show a slow degradation rate and good mechanical properties [5,6]. That is compared to natural polymers, which show biodegradability, bioactivity, and biocompatibility, such as collagen and alginate [7]. 

Cellulose acetate (CA) is a semi-synthetic polymer [8]. CA is non-mutagenic, non-toxic, non-carcinogenic, and non-immunogenic [2]. These properties are why they are widely investigated in bone tissue engineering articles [9,10]. Therefore, it is an excellent biocompatible polymer. In addition, its mechanical properties, such as tensile stress, are good, despite its low crystallinity [11,12]. This property means that CA can simulate the bone tissue microenvironment when used as a scaffold. H. Tan et al. prepared nanofibers based on CA as a scaffold, and they reported that it showed high biocompatible properties. The cell viability exceeded 100% towards osteoblast cells [9]. A. Tsiapla et al. studied CA loaded with dexamethasone and reported that the degradability reached around 25% after 150 days. In addition, dexamethasone is completely released after around 180 days. Further, cell viability reached around 150% after 5 days [13].

Metal oxide nanoparticles have gained attraction and considerable interest due to their distinct and unique physical and chemical features. They are widely used in various applications such as photocatalysis, textile, heavy metal removal, gas sensing, and biomedical applications. Additionally, bacteria that develop themselves against conventional drugs become difficult-to-treat infections. This challenge can be treated with metal oxide nanoparticles, showing good, promising results in the literature. Silver oxide (Ag_2_O) is one of the metal oxides used in many applications, such as data storage devices, photocatalysis, photovoltaic cells, sensors, and medical applications. In addition, being used as an antibacterial agent in composites due to its desirable properties. A. Rajabi et al. prepared nanocomposites containing Ag_2_O and CuO. They reported the antibacterial activity against *E. coli* and that the nanoparticles had a positive charge, while the bacteria had a negative charge. Therefore, electrostatic interaction occurs, leading to oxidation stress and bacterial death [14]. Further, A. Rajabi et al. prepared Ag_2_O/polyethylene terephthalate and reported that the inhibition zone against *E. coli* reached 14 ± 0.2 mm [15]. That indicates the successful use of Ag_2_O as an antibacterial agent against *E. coli*.

Zinc is one of the essential elements in the human body that serves in immunity, metabolism, and cell growth [16,17]. Zinc and sulfur aim to heal wounds in the skin and exhibit inflammation effects [18]. Zinc sulfide (ZnS) is in the film composition, which can directly affect cell growth and biocompatibility. K. Sharma et al. reported the ability of ZnS to purify water as a photocatalysis material [19]. Therefore, ZnS can be used to enhance the ability of Ag_2_O to kill bacteria and improve biocompatibility. 

Graphene oxide (GO) is a graphene derivative material, an extraordinary material formed of a single layer of carbon atoms in sp^2^ hybridization in a honeycomb lattice [20]. GO has functional groups such as carboxylic acids, epoxy, and hydroxyl. GO has hydrophilic properties, high drug-loading capacity, size, and shape that can be controlled, high mechanical properties, and a large surface area [21]. The growth factors that are essential formation, migration, and differentiation can be transported using GO [22]. Therefore, GO can play a critical role in biomedical applications [23]. M. Tavakoli et al. prepared a nanocomposite containing GO/chitosan/polymethyl-methacrylate, and they found that the existence of GO improved the biological response. The cell viability reached around 100% after 3 days of incubation towards MG-63 cells [24]. S Purohit et al. synthesized a nanocomposite of alginate, gelatin, and GO. They reported that GO directly affected the mechanical properties, increasing from 30 MPa to 55 MPa. Further, the cell attachment after 8 h enhanced from around 50% to about 70%, and in the same way, the proliferation increased from 130% to 250% on the seventh day [25]. The combination of GO, ZnS, Ag_2_O, and CA can present better properties when combining them.

Antibacterial activity is essential to prevent antibacterial invasion and decrease inflammation. T. Li et al. designed wound dressing materials consisting of a silk fibroin/polycaprolactone/polyvinyl alcohol directional moisture transport composite sheet filled with antibacterial drug-loading microspheres [26]. The results showed that the antibacterial activity of the sheets against *S. aureus* and *E. coli* is 98.79% and 95.39% [26]. Y. Zhang et al. prepared, using wet spinning, a multipurpose sodium alginate (SA)@ urushiol fiber with specific antibacterial, acid corrosion resistance, and flame-retardant characteristics [27]. The results showed that the SA@ urushiol fiber had a 99.99% effective bactericidal rate against *S. aureus*. On the other hand, urushiol had a delayed impact on *E. coli*’s ability to multiply at low concentrations, perhaps as a result of the substance’s structure [27], and it wasn’t until the urushiol level reached 3 wt % that it had a bacteriostatic effect, hitting 72.73% [27]. Cytotoxicity and cell viability are tests to measure the viable, normal cells, while antibacterial activity is a test to study the behavior of the scaffold against bacterial invasions, which could cause skin inflammation.

In this study, a combination of Ag_2_O, ZnS, and GO is encapsulated in CA film to be utilized as a novel wound dressing material for wound healing application. The main reason for choosing Ag_2_O is due to its usage in different medical applications and its superb antibacterial activity. On the other hand, ZnS is selected as Zn has many desired properties in biological applications. Zn aids in cell growth and serves the immune system. Sulfur and zinc both work to reduce inflammation and heal skin wounds. Further, GO is supposed to increase the hydrophilic nature of CA and aid in cell adhesion. Moreover, CA has good biocompatibility, and it is a non-toxic material for the human body. CA serves as a matrix for the previous drugs. XRD is used to investigate the crystallinity and crystal symmetry of the used materials, while FTIR is used to describe the functional groups of the modified films. On the other hand, SEM and EDX are used to study the surface morphology and particle size of the films and to determine the percentages of the selected area, respectively. Further, the contact angle test is measured to study the behavior of the films with the water drops, while the UV-Vis measurement is tested to measure the transparency of the films and the dispersion of the additives. Furthermore, the TGA test is used to study the thermal stability of the films. Additionally, the antibacterial activity and cell viability are tested for the samples. All these measurements and tests are carried out to understand the behavior of the prepared films with the wounded area. This combination is thought to be a suitable material to fit with wound healing applications.

## 2. Materials and Methods

### 2.1. Materials

Silver oxide (Ag_2_O), zinc nitrate (Zn(NO_3_)_2)_, sodium sulfide (Na_2_S), and sodium hydroxide (NaOH) were purchased from Sigma-Aldrich Co., St. Louis, MO, USA. The human fibroblasts cell line was used under culturing conditions in Dulbecco’s modified Eagle’s medium (DMEM, Gibco, Waltham, MA, USA) to inspect cell viability. Cellulose acetate (CA) Mw = 30,000 g/mol was purchased from ACROS. Acetone was obtained from Merck, Darmstadt, Germany. 

### 2.2. Experimental Methods

The compositional materials (Ag_2_O, ZnS, GO) were obtained in the powder phase and then incorporated into the polymeric phase of CA. An amount of 5 g of Zn(NO_3_)_2_ was dissolved in deionized water (DIW), and 5 g of Na_2_S was dissolved in another beaker. Then, S solution was added slowly into the Zn solution, and NaOH was dropped into the mixed solution to obtain ZnS precipitated gel. The obtained gel was dried in a furnace to obtain the powder. The polymeric film samples were prepared as follows: The first sample was prepared by dissolving 2 g of CA powder in 20 mL of acetone under a magnetic stirrer for 15 min. The second sample was prepared by dissolving the 2 g of CA in 20 mL of acetone under a magnetic stirrer, then about 0.25 g of Ag_2_O was dropped in the CA bottle and stirred for 20 min to disperse the solution. Further, the third sample was prepared by dissolving 2 g of CA in 20 mL of acetone in the bottle under stirring, then 0.25 g of ZnS was added to the solution bottle slowly and stirred for 20 min to be well dispersed in the solution. The fourth sample was prepared by filling the bottle with 20 mL of acetone and dropping 2 g of CA slowly into the bottle under magnetic stirring for 15 min to dissolve the polymer, then about 0.125 g of Ag_2_O was added to the solution and dispersed after 20 min of stirring, and 0.125 g of ZnS was added to the same solution and dispersed after 20 min of stirring. Finally, the last sample was prepared by dissolving about 2 g of CA in 20 mL of acetone in a bottle, then Ag_2_O, ZnS, and GO were added separately to the CA solution, and every one of them was dispersed for 20 min in the CA solution. The final step to obtaining CA films was that each sample was poured into a Petri dish with a diameter of 14 cm, and the diameter of the CA films was 2 mm; after that, the sample was placed in drier francs at 40 °C until the solvent evaporated and CA films formed.

### 2.3. Characterization Methods

X-ray diffractometer was used to obtain the patterns of XRD (Malvern PANanalytical X′Pert Pro, Cu k_α1_ = 1.5404, Malvern, WR14 1XZ, UK). Morphological analysis was conducted by scanning electron microscope (SEM) with an operating voltage of 20–30 kV (QUANTA-FEG250, Kolkata 700106, WB, India). Through a wavenumber range of 4000–400 cm^1^, Fourier-transform infrared (FTIR) spectra were measured by using an FTIR spectrometer (Perkin-Elmer 2000 Akron, OH, USA). The surface morphology of the materials and EDX analysis were investigated using a scanning electron microscope (SEM) (QUANTA-FEG250, Kolkata 700106, WB, India).

### 2.4. The Water Contact Angle

A customized system was used to detect the angle of water droplets on the sample. On 2 cm^2^ of a sample, the water droplets were carefully dropped. After positioning the sample upon that stand in front of the camera, the image was taken by (HiView). Three experiments were made for each sample to determine the contact angle of each one. The droplet diameter was 4 mm ± 1.4.

### 2.5. Thermal Gravimetric Analysis (TGA)

With an airflow rate of 100 mL/min, TGA was performed in a (DTG-60H Shimadzu) analyzer from room temperature (R. T.) up to 1173 K. There was a 10 K/min heating rate.

### 2.6. UV-Vis Spectrophotometer

UV–visible (UV-Vis) spectroscopy (METASH, Shanghai, China) was employed to examine the optical characteristics of the films from 400 to 800 nm. The system was calibrated to air. About 2 × 2 cm^2^ of each film was placed in front of the UV lamp and tested.

### 2.7. Cytotoxicity Test

Human lung cells were used under culturing conditions in Dulbecco’s modified Eagle’s medium (DMEM, Gibco, ThermoFisher Scientific, Gillingham, UK) to inspect cell viability. Cells with a density of 5 × 10^3^ (cells/cm^2^) were cultured on the composites through 24-well plates and then incubated at 37 °C. After three days of incubation, media were removed, and MTT (3-(4,5-dimethylthiazol-2-yl)-2,5-diphenyltetrazolium bromide) was injected into each well, then cell viability was detected through an optical analyzer (The Vi-Cell XR Cell Viability Analyzer, Beckman Coulter, Indianapolis, IN, USA).

### 2.8. Antibacterial Measurement

The antibacterial behavior of the films was measured by diffusion mechanism. The antibacterial behavior was tested against 2 types of bacteria: *Staphylococcus aureus* (*S. aureus*) and *Escherichia coli* (*E. coli*). About 0.055 mg from each film was immersed in 2 mL of DIW for 24 h. Each film was immersed in 2 mL of water separately. After that, the inhibition zone was calculated for each film.

## 3. Results and Discussions

### 3.1. XRD

**Figure 1:**
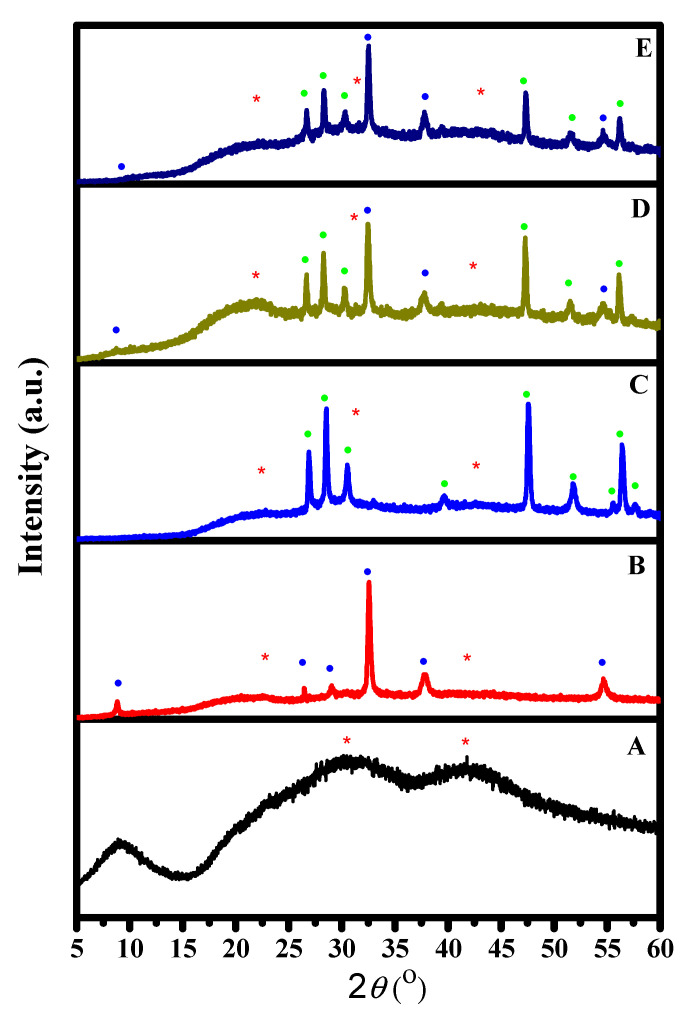
X-ray diffraction (XRD) pattern of different composites (**A**) CA, (**B**) Ag_2_O/CA, (**C**) ZnS/CA, (**D**) Ag_2_O/ZnS/CA, and (**E**) Ag_2_O/ZnS/GO/CA: (**•** blue dot) Ag_2_O, (**•** green dot) ZnS, and (*) CA.

The XRD technique was used to investigate the crystal symmetry and crystalline nature of the compositions, as shown in Figure 1. The patterns are exhibited in Figure 1. However, in the pure CA in Figure 1A, the pattern shows diffuse peaks, which indicate the amorphous nature at around 30.9° and 41.6°. There is also a peak at around 9.2°, which is less broadening than the others, indicating the crystalline nature of CA. Therefore, this explains why CA is considered a semicrystalline polymer. Further, the low signal-to-noise ratio indicates the low crystallinity of the polymer. These observations agree with previous studies [28,29,30]. Figure 1B shows cubic crystal symmetry with lattice parameters around 0.472 nm (JCPDS File No. 75e1532) [31]. Further, the most characteristic peaks of Ag_2_O can be determined at 32.5°, 37.8°, and 54.6°. The mentioned peaks belong to the crystal planes of (111), (200), and (220) [32]. From Figure 1C, ZnS hexagonal symmetry can be determined with peaks at 26.9°, 28.5°, 30.5°, 47.6°, and 56.4° [33,34]. These peaks belong to (100), (002), (110), and (112), respectively [33]. The composition of Ag_2_O/ZnS/GO/CA, as shown in Figure 1E, contains three phases, which belong to Ag_2_O, ZnS, and CA, while the peaks of GO cannot be observed due to the low quantity of GO relative to the total composition.

### 3.2. FTIR

**Figure 2:**
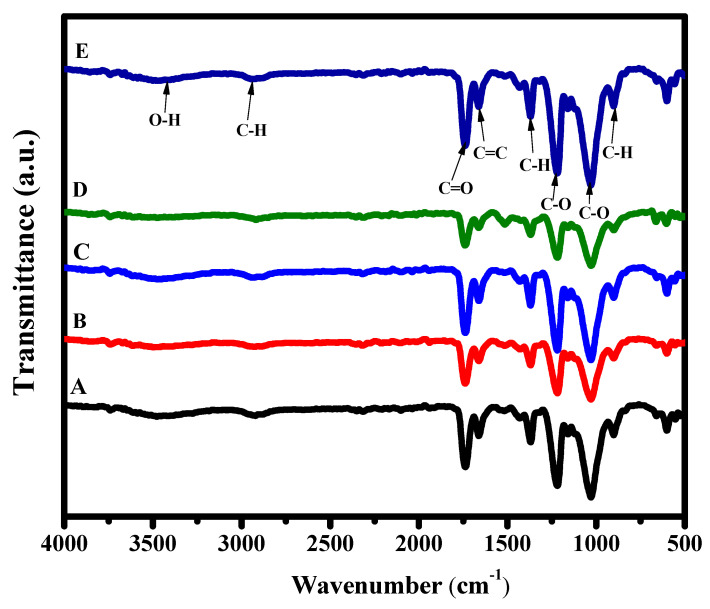
Fourier-transform infrared (FTIR) spectra for detecting the transmittance spectra of the different composites (**A**) CA, (**B**) Ag_2_O/CA, (**C**) ZnS/CA, (**D**) Ag_2_O/ZnS/CA, and (**E**) Ag_2_O/ZnS/GO/CA.

FTIR spectroscopy was used to determine the functional groups in the prepared compositions and is illustrated in Figure 2 and Table 1. It can be observed that there is a band at around 1738.44 cm^−1^, which belongs to C=O stretching vibration (carbonyl group). Two C-O bands can be determined at 1211.88 and 1027.16 cm^−1^, respectively [35]. These groups confirm the existence of the ester group (O=C-O) in cellulose acetate [36]. The mentioned peaks of C-O have asymmetric vibrations of the (-C-O) that left the oxygen ester (higher wavenumber) and (O-C-), which is to the right of the oxygen ester (lower wavenumber). Further, the acetyl group also has a characteristic band of C-H due to the C-CH_3_ (alkane) group, which can be determined at 1368.24 cm^−1^ [37,38]. Moreover, the band at 901.69 cm^−1^ belongs to the bending vibrational mode of C-H [39]. Furthermore, the O-H band originated from the adsorbed water molecules and can be detected, with a broad but fragile band, at around 3431.03 cm^−1^ [40]. It can be observed that after the addition of GO, there are bands that have more intensity, such as 1027.30, 1212.02, and 1746.30 cm^−1^. These bands become more robust due to the existence of alkoxyl (HO-CH_3_), epoxy (C-O-C), and carboxyl (O=C-OH) groups, which confirm GO incorporation [41]. It can be observed that there are two weak bands around 485 and 1380.2 cm^−1^ that are due to Ag-O [42]. On the other hand, it can be seen that ZnS shares a peak with CA at 1027.16 cm^−1^ [43]. The two weak peaks at 485 and 677.2 are attributed to Zn-S cm^−1^ [43]. The peaks of ZnS and Ag_2_O could be due to their very low concentrations compared to CA film.

### 3.3. SEM/EDX Analysis

**Figure 3:**
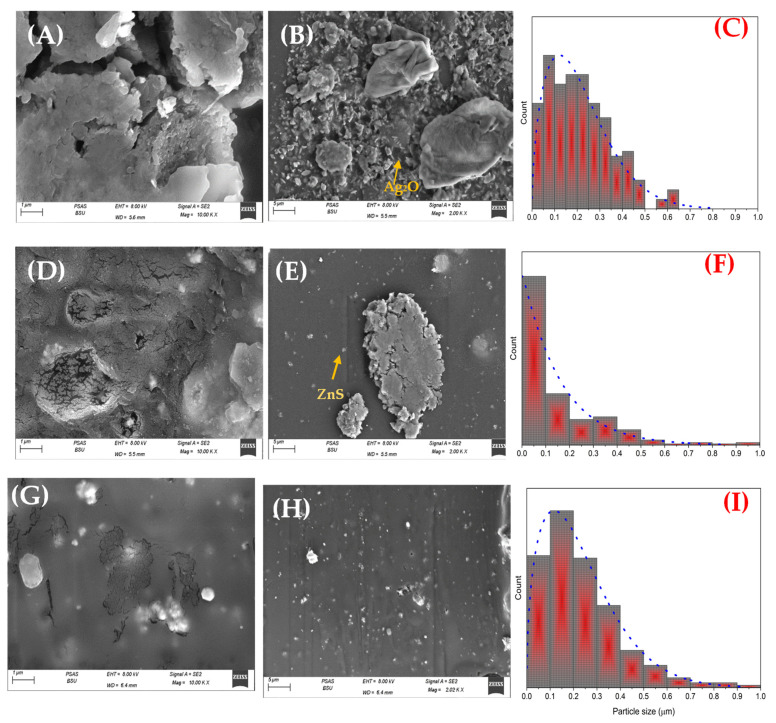
Scanning electron microscope (SEM) micrographs of (**A**) Ag_2_O/CA with 1 µm scale bar, (**B**) Ag_2_O/CA with 5 µm scale bar, (**C**) particle distribution of Ag_2_O/CA, (**D**,**E**) Ag_2_O/ZnS/CA with 1 and 5 µm scale bar and particle distribution, (**F**) particle distribution of Ag_2_O/ZnS/CA, (**G**,**H**) Ag_2_O/ZnS/GO/CA with 1 and 5 µm scale bar and particle distribution, and (**I**) particle distribution of the embedded Ag_2_O/ZnS/GO on CA film.

**Figure 4:**
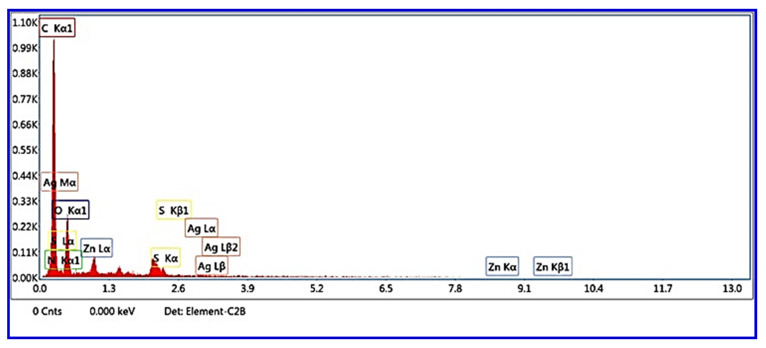
Energy-dispersive X-ray (EDX) spectrum of Ag_2_O/ZnS/GO/CA.

A scanning electron microscope was used to study morphology and changes in size distribution and shape. The size and shape are essential parameters in biomedical applications that can reflect the self-assembly of particles. The magnification micrograph (10kX) is demonstrated in Figure 3A and shows a polymeric membrane of CA covered with particles of Ag_2_O. These particles have a wide-size distribution from a few nanometers to a hundred nanometers. In addition, more information can be collected from lower magnification (2kX) in Figure 3B, such as the irregular shape. Still, most are rod morphs for the nanoparticles and a rocky texture for large, aggregated particles. Further, Figure 3C shows the size distribution, which is in the range of 19–648 nm 333.5 ± 56.3 nm with an average size of 216 nm. Moreover, the addition of ZnS to the nanocomposite directly affected the morphology, as can be seen in Figure 3D,E. Due to the broader distribution of ZnS particles, more nanoparticles are added to the nanocomposite. Furthermore, the porosity of CA is obvious in this sample, which is a critical factor in biomedical applications. The average particle size is around 158 nm, and the distribution in the range of 12–991 nm means, 560 ± 90.6 nm smaller and larger particles are incorporated by adding ZnS. The addition of GO nanosheets and their effect on the morphology can be observed in Figure 3G–I. It can be seen that the CA film is covered with particles of Ag_2_O/ZnS with a nanosheet of GO. That led to less distribution width than the composition of Ag_2_O/ZnS/CA, where the sizes are in the range of 18–915 nm with an average size of 235 nm. It is very hard to recognize the dopants in the film due to their low concentration compared to the concentration of the film; the maximum concentration of the embedded dopants is 12.5 wt % from the total percentage of the film, while CA is 88.9 wt %. Additionally, in the final sample, the weight percent of CA, Ag_2_O, ZnS, and GO are added to CA with the following percentages 88.9/4.44/4.44/2.22 wt %, respectively. The difference between the particles can be recognized from the other works of the literature. The weight ratio of CA to the particles is CA, Ag_2_O, ZnS, and GO 88.9/4.44/4.44/2.22 wt %, respectively. So, it is very hard to recognize the particles as they have a low ratio compared to CA and are embedded in the film. Moreover, the EDX investigation was based on SEM micrographs, and it is shown in Figure 4 that the elements of CA and GO can be detected by determining C and O, which form 94.21% of the film. In addition, the high content of oxygen is due to its sharing with Ag_2_O, GO, and CA. Moreover, Table 2 shows that the Zn, S, and Ag can also be determined, consisting of Ag_2_O and ZnS. The very low percentage of nitrogen may be due to the organic cellulose acetate. 

### 3.4. Contact Angle

The contact angle measurement is a critical factor in film properties due to its importance in determining the hydrophobicity and hydrophilicity behavior. The ideal hydrophilic surface has a contact angle of 0°, and when the angle increases, the hydrophobic character of the film increases [45]. The measurements are exhibited in Table 3, and it can be seen that pure CA has a contact angle of 48.04° ± 1.59°. The addition of Ag_2_O led to enhancement in the spreading of the water droplet, where the contact angle decreased to 46.12° ± 2.65°. Furthermore, adding ZnS enhanced the hydrophilicity, and the angle reached 45.46° ± 2.50°. Moreover, the Ag_2_O/ZnS/CA composition shows the highest hydrophilic behavior, and the contact angle reached 26.28° ± 2.12°. The hydrophilic behavior decreased by adding GO to the composition to around 34.04° ± 3.24°. The increase in the hydrophilic nature of CA due to the presence of additives would enhance the surface interaction with body fluids. This means that the skin moisture is higher, and cell proliferation and adhesion are higher. Consequently, more cells can proliferate and grow on the surface of the film. The decrease in contact angle may be due to the increase in roughness as a result of the addition of the chosen particles in the film. The increase in surface roughness would aid in the wettability of the surface, which will decrease the angle. The hydrophilicity of the film was boosted by the reactive hydroxyl groups in the GO, particularly the hydroxyl OH group on the film’s surface. The density of hydrophilic groups present on the surface of this film also reduces the energy of the water interaction [46]. The positive effects of encapsulated GO on film hydrophilicity were in line with the results of other studies of a similar kind [46].

### 3.5. TGA

**Figure 5:**
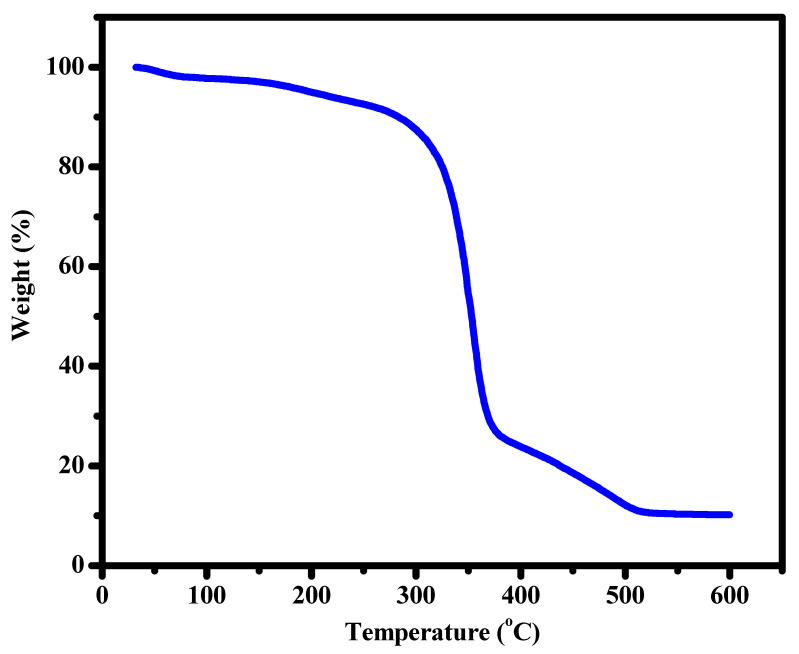
The TGA analysis for the final Ag_2_O/ZnS/GO/CA composite from room temperature to 600 °C.

Thermal stability is essential for any film owing to the flexibility of biomaterial inside the human body under different temperatures and thermal conditions. However, the TGA analysis is illustrated in Figure 5, and it can be noticed that the degradation of Ag_2_O/ZnS/GO/CA started at around 300 °C. The composition lost about 80% of its weight at about 370 °C, and at 600 °C, it lost about 90% of its weight. This is due to the acetate group loss, dehydration, and depolymerization of CA, and the remaining 10% at the end are Ag_2_O, ZnS, and GO. However, that means the composition is stable under the thermal circumstances humans can expose. The final sample was chosen for the TGA test as it is the film that contains all materials. The TGA curve was studied for the combination as it is the sample that exhibited the most favorable properties in the other tests. Thermal stability is an important property to study the behavior of the film from room temperature to 600 °C. According to the TGA curve, three stages can be studied. TGA is useful as the injured area might be exposed to a high temperature accidentally. So, it is important to know the behavior of the film with different temperatures. This study might allow us to find other applications for the film.

### 3.6. Cytotoxicity

**Figure 6:**
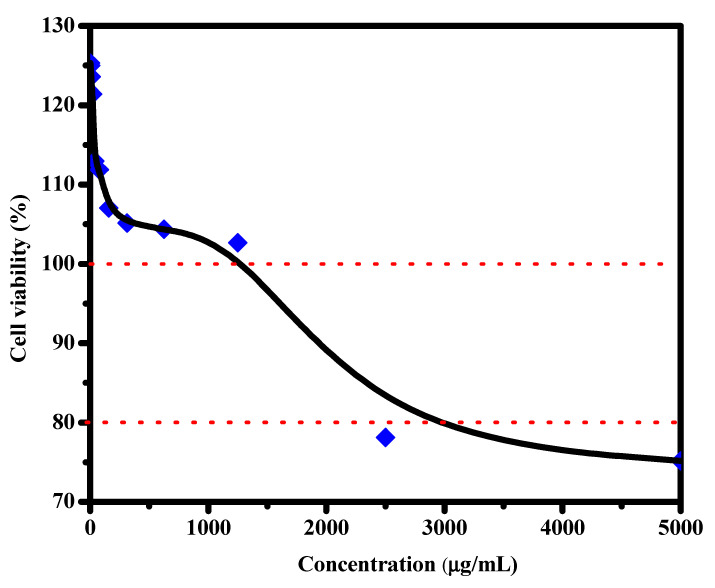
Cell viability of normal lung cells upon different Ag_2_O/ZnS/GO/CA concentrations.

The normal lung cell line was used to investigate the biocompatibility and cytotoxicity of the prepared film. The initial concentration was 5000 µg/mL. The results are demonstrated in Figure 6; it can be noticed that the small concentration of around 2.44 µg/mL showed a cell viability of 125.31%. By increasing the concentration, the viable cells decreased but at a low rate, where 102.66% of cells were viable at 1250 µg/mL. The highest tested concentration was 5000 µg/mL, which records relatively high cell viability with a value of 75.16%. These results indicate the biocompatibility of Ag_2_O/ZnS/GO/CA, which contains CA and GO, known as biocompatible materials. Moreover, the morphology of the composition, such as the film’s porous structure, led to reaching the cells’ nutrients and oxygen [47]. Therefore, more cells are viable with the existence of the film in addition to the enhancement in the hydrophilic behavior. The reactive hydroxyl groups of the GO, in particular, the hydroxyl (O-H) group on the film’s surface, increased the hydrophilicity of the film. Additionally, the density of hydrophilic groups on this film’s surface minimizes the energy of the water contact [46]. The favorable effects of encapsulating GO on film wettability are consistent with findings from earlier investigations of the same kind [46]. The addition of GO to the surface of the film might also increase the surface roughness, which plays a significant role in increasing the hydrophilicity, which would increase cell adhesion on the scaffold and cell generation. The porosity in the film aided in the growth and generation of cells, which explains the growth of the cells until reaching 125.31% viable cells. In addition, the enhancement in the hydrophilic behavior of CA occurred as a result of the addition of the mentioned drugs in the CA film.

Cytotoxicity and cell viability are tests to measure viable normal cells, while antibacterial activity is a test to study the behavior of the scaffold against bacterial invasion, which could cause skin inflammation.

### 3.7. Uv-Vis

**Figure 7:**
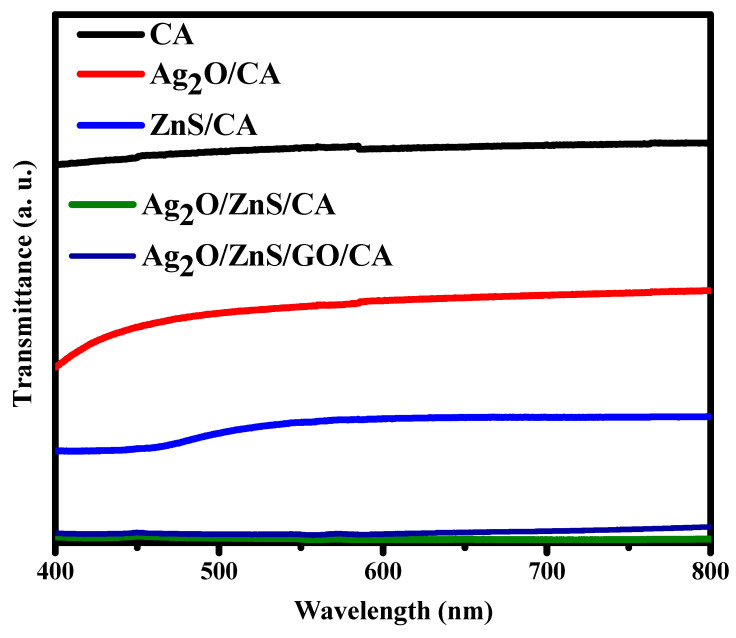
UV-Vis transmittance measurement for all composites.

Concerning the optical properties or the transparency of the prepared composition of Ag_2_O/ZnS/GO/CA, less transparency means less light can pass through the film. The degree of the dispersed particles is one of the main factors that control this optical behavior. Pure CA shows relatively high transmittance photons at around 75%. The addition of Ag_2_O decreased that transparency to about 45%, as shown in Figure 7. Further, ZnS decreased that behavior to about 20%, and this indicates the ability of ZnS to prevent light from being passed through the film. Moreover, adding Ag_2_O/ZnS and Go into the CA phase blocked the photons from transmitting with a transmittance value lower than 5%. This behavior is due to the agglomerated particles detected by the SEM investigation. The larger particles are shown in distribution particle sizes, which directly affect the optical properties by blocking the photons from being transmitted through the sample. The optical properties are essential as the transparency of the films means that the particles are well dispersed on the film surface, which is a targeted result. When the light falls on the surface of the film and particles, free electrons and charge carriers are generated, which could aid in the degradation of the bacteria and inhibit inflammation. Additionally, it could play a key role in sterilizing the wounded area.

### 3.8. Antibacterial

**Figure 8:**
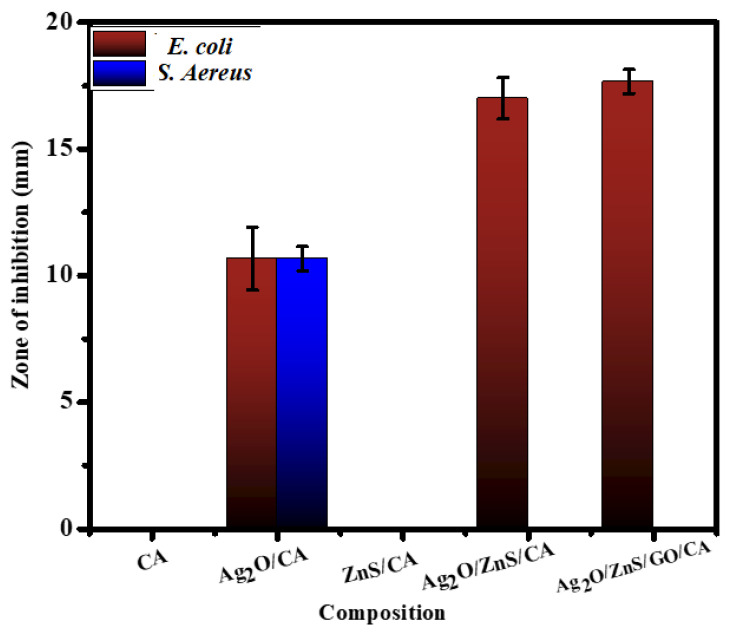
Antibacterial activity of the different composites after 24 h of exposure against *E. coli* and *S. aureus*.

The bacterial growth was determined and is illustrated in Figure 8. It can be clarified that CA and ZnS/CA have no antibacterial activity against both strains. In this regard, *S. aureus* is only sensitive to Ag_2_O/CA and showed 10.7 ± 0.5 mm as a zone of inhibition. The other films showed no antibacterial activity against *S. aureus*. Further, Ag_2_O/CA showed a zone of inhibition with a value around 10.7 ± 1.2 mm against *E. coli*. The addition of ZnS developed that activity and reached 17.0 ± 0.8 mm. Moreover, adding GO slightly enhanced the antibacterial behavior, and the zone of inhibition reached 17.7 ± 0.5 mm. The antibacterial activity of Ag ions has been reported in previous studies, affecting the cell membrane; consequently, *E. coli* bacteria cannot establish protection, thereby leading to cell death [48,49,50]. In the case of Ag_2_O/ZnS/GO, the weight percentage of each dopant was lowered; for example, by comparing the percentage of Ag_2_O in the second and final samples, we could recognize that the weight percentage of Ag_2_O decreased from 11.11 wt % to 4.44 wt % from the total weight of the film. So, the effect of the Ag_2_O on killing bacteria is lowered or not found.

## 4. Conclusions

Silver oxide (Ag_2_O), zinc sulfide (ZnS), and graphene oxide (GO) were separately prepared and combined with cellulose acetate (CA). Structural, compositional, elemental, and morphological investigations were conducted. In addition, the biological response was investigated in vitro. XRD showed the cubic and hexagonal symmetries of Ag_2_O and ZnS in addition to the low crystallinity of CA. Fourier-transform infrared spectroscopy investigated and detected the epoxy, hydroxyl, and carboxylic functional groups in the Ag_2_O/ZnS/GO/CA composition. Further, the morphological investigation using scanning electron microscopy (SEM) showed that the average particle size reached 158 nm with a distribution range of 12–991 nm. In addition, the contact angle decreased from 48.04° ± 1.59° to 34.04° ± 3.24° and, consequently, the hydrophobicity. The transmittance efficiency decreased from around 75% to 5%, and the composition was thermally stable until 300 °C. Moreover, the antibacterial activity enhanced and reached 17.7 ± 0.5 mm against *E. coli* bacteria. Additionally, Ag_2_O/ZnS/GO/CA composition showed a cell viability value of 125.31% at 2.44 µg/mL and reached 75.16% at 5000 µg/mL. The low contact angle, high antibacterial activity, high viable cells, cell generation, and good dispersion of the additives in the CA films largely support this film’s use in wound healing applications.

## Figures and Tables

**Table 1:** The detected bands by FTIR technique and their assignments.

CA (cm^−1^)	Ag_2_O/CA (cm^−1^)	ZnS/CA (cm^−1^)	Ag_2_O/ZnS/CA (cm^−1^)	Ag_2_O/ZnS/GO/CA (cm^−1^)	Assignment	Refs
-	-	485	485.2	457.6	Zn-S	[43]
-	561.8	-	561.9	562	Ag-O	[42]
-	-	677.2	671.5	676	Zn-S	[43]
901.69	901.03	901.33	901.25	901.38	C-H	[39]
1027.16	1027.17	1026.90	1027.53	1027.30	C-O Zn-S	[36,43]
1211.88	1212.74	1212.55	1212.36	1212.02	C-O	[36]
1368.24	1367.76	1368.30	1368.11	1367.86	C-H	[37]
-	1380.2	-	1378.2	1377.9	Ag-O	[42]
1663.46	1662.89	1662.58	1663.23	1663.25	C=C	[37]
1738.44	1743.94	1746.07	1744.29	1746.30	C=O	[37]
2901.12	2922.70	2912.33	2922.70	2931.48	C-H	[44]
3431.03	3451.77	3462.15	3431.03	3422.25	O-H	[36]

**Table 2:** The quantification analysis of EDX is based on SEM micrographs.

Element	Atomic (%)
C K	70.78
N K	4.19
O K	23.43
S K	0.41
AgL	0.81
ZnK	0.38

**Table 3:** The contact angle of all composites with the error bars was estimated by repeating the experiment 3 times.

Sample	Angle	Stander Deviation
CA	48.04	1.59806
Ag_2_O/CA	46.12	2.65872
ZnS/CA	45.64	2.50316
Ag_2_O/ZnS/CA	26.285	2.12839
Ag_2_O/ZnS/GO/CA	34.045	3.24562

## Data Availability

The raw/processed data generated in this work are available upon request from the corresponding author.
